# Endothelial Dysfunction of Patients with Peripheral Arterial Disease Measured by Peripheral Arterial Tonometry

**DOI:** 10.1155/2016/3805380

**Published:** 2016-10-18

**Authors:** Kimihiro Igari, Toshifumi Kudo, Takahiro Toyofuku, Yoshinori Inoue

**Affiliations:** Division of Vascular and Endovascular Surgery, Department of Surgery, Tokyo Medical and Dental University, 1-5-45 Yushima, Bunkyo-ku, Tokyo 113-8519, Japan

## Abstract

*Objective*. Endothelial dysfunction plays a key role in atherosclerotic disease. Several methods have been reported to be useful for evaluating the endothelial dysfunction, and we investigated the endothelial dysfunction in patients with peripheral arterial disease (PAD) using peripheral arterial tonometry (PAT) test in this study. Furthermore, we examined the factors significantly correlated with PAT test.* Methods*. We performed PAT tests in 67 patients with PAD. In addition, we recorded the patients' demographics, including comorbidities, and hemodynamical status, such as ankle brachial pressure index (ABI).* Results*. In a univariate analysis, the ABI value (*r* = 0.271, *P* = 0.029) and a history of cerebrovascular disease (*r* = 0.208, *P* = 0.143) were found to significantly correlate with PAT test, which calculated the reactive hyperemia index (RHI). In a multivariate analysis, only the ABI value significantly and independently correlated with RHI (*β* = 0.254, *P* = 0.041).* Conclusion*. This study showed a significant correlation between RHI and ABI. The PAT test is a useful tool for evaluating not only endothelial dysfunction but also the hemodynamical state in patients with PAD.

## 1. Introduction

The impairment of endothelial function, which predisposes one to thrombosis, leukocyte adhesion, and smooth muscle cell proliferation, is centrally involved in the atherosclerotic disease process [1, 2]. Therefore, the evaluation of endothelial dysfunction is useful for treating atherosclerotic diseases [3].

Recently, several noninvasive methods of assessing the endothelial dysfunction have been developed. Flow-mediated dilatation (FMD) is one such particularly useful technique, where the endothelial function is evaluated via ultrasound measurement of the brachial artery diameter during reactive hyperemia [4]. FMD detects an indicator of nitric oxide-mediated endothelium-dependent vasodilatation; therefore, FMD might be influenced by the endothelial dysfunction [5]. Patients with peripheral arterial disease (PAD) have shown a reduction in FMD, and ischemia by acute exercise has also been found to lead to a reduction [6, 7]. Therefore, despite how commonly FMD is used to evaluate the endothelial dysfunction, this technique has not yet been wholly endorsed by the scientific community as a standardized measuring method and a standardized index for evaluating the endothelial function [8, 9].

Peripheral artery tonometry (PAT) is another noninvasive test for evaluating the endothelial function. In PAT, the pulse wave amplitude is assessed before and during reactive hyperemia, which is induced by occluding blood flow of the brachial artery using an inflatable cuff. The calculated index (reactive hyperemia index; RHI) between the flow in the arm with reactive hyperemia and the control arm represents a measure of the endothelial function [10, 11]. PAT might produce more reproducible results than FMD, the results of which might vary among ultrasound sonography technicians.

We herein evaluated the endothelial dysfunction of PAD patients using PAT and investigated the relationship between the endothelial dysfunction and various parameters.

## 2. Patients and Methods

### 2.1. Patient Selection

We recruited 67 patients with PAD due to atherosclerosis, from the outpatient clinic at Tokyo Medical and Dental University Hospital between May 2014 and July 2015. All of the recruited patients provided their informed consent, and approval was obtained from our Institutional Review Board for a retrospective review of the patients' medical records and images.

We diagnosed PAD using such modalities as computed tomography angiography, magnetic resonance angiography, duplex ultrasound sonography, or digital subtraction angiography based on the presence of >50% vessel stenosis due to the lesions in the lower limbs. We excluded those patients who had received a recent intervention for PAD or coronary artery disease (CAD) (<6 months) and who had a recent history (<3 months) of unstable angina, myocardial infarction, or cerebrovascular disease (CVD), including stroke, decompensated heart failure, malignant disease, and systemic inflammatory disease, such as infectious diseases.

We obtained the patients' demographics, medications, and medical histories using a dedicated database, retrospectively. The patients' medical records were reviewed as described below. Hypertension was defined as a systolic blood pressure of >130 mmHg, a diastolic blood pressure of >80 mmHg, or a history of treatment for hypertension. Dyslipidemia was defined as a serum LDL cholesterol level of >140 mg/dL, an HDL cholesterol level of <40 mg/dL, a triglyceride level of >150 mg/dL, or a history of treatment for dyslipidemia. CAD was defined as the presence of angina pectoris or myocardial infarction or both, as documented on coronary angiography or based on a history of any revascularization procedures of the coronary arteries. CVD was defined as a history of stroke, transient ischemic attacks, carotid artery revascularization, or cerebral hemorrhage. Chronic kidney disease (CKD) was defined as an estimated glomerular filtration rate < 60 mL/min/1.73 m^2^. Diabetes mellitus (DM) was defined as a fasting blood glucose > 126 mg/dL, hemoglobin A1c > 6.5%, or being medicated with antidiabetes drugs. The severity of PAD was assessed by measuring the ankle brachial pressure index (ABI), which was calculated as the ankle systolic blood pressure divided by the brachial systolic blood pressure using the VasoGuard P84™ system (SciMed Ltd., Bristol, UK). The ABI for each patient was set as the lower of the ABI levels for each leg or nonrevascularized limb.

### 2.2. Laboratory Measurements

After the patients had fasted for at least 12 hours, a venipuncture was made in the superficial vein of the upper limb for the collection of the blood samples. Complete blood cell counts, including hemoglobin; biochemistry examinations, including low-density lipoprotein and C-reactive protein; and coagulation tests, including fibrinogen, were conducted via standard laboratory methods.

### 2.3. Endothelial Function Test

The endothelial function was measured by PAT using an EndoPAT 2000 device (Itamar Medical Ltd., Caesarea, Israel), which assessed the RHI as a measure of the microvascular endothelial function. Patients refrained from smoking, drinking caffeine-containing beverages, and eating for 12 hours before the PAT measurement. The patients also discontinued all drugs for 18 hours before the measurement and any food for 12 hours before the measurement.

The patients were placed in a supine position with a specially designed finger probe on the index finger of each hand and a pressure cuff on one upper arm in a quiet room at a constant temperature of 20°C. After continuous recording of the PAT signal from both fingers during a 10-minute baseline period, the blood pressure cuff on the study arm was inflated to suprasystolic pressure for 5 minutes. The inflated cuff was then deflated, and recording of the PAT signal continued for 10 minutes. The pressure changes reflecting the pulse amplitude both before the inflation of cuff and after the deflation were transmitted to a computer, and the RHI was calculated automatically [12].

### 2.4. Statistical Analysis

The continuous variables are expressed as the mean ± standard deviation (SD), and the categorical variables are expressed as the frequencies and percentages. For the categorical variables, the significance was evaluated by *t*-test, and the correlations for the continuous variables were obtained using Pearson correlation, which reflected the degree of relationships between variables. These variables were evaluated in a univariate model, and variables with *P* < 0.15 were then entered into a multivariate regression analysis. *P* < 0.05 was defined as statistical significance. The statistical analyses were performed using the Stat View version 5 software program (Abacus Concept Inc., Berkley, CA, USA).

## 3. Results

### 3.1. Patient Demographics (Table 1)

We evaluated 67 patients (50 males and 17 females) with PAD in this study. The mean age was 73.1 ± 8.1 years, and the mean body mass index was 22.3 ± 2.5 kg/m^2^. The documented comorbidities were smoking history (76.1%), hypertension (83.6%), dyslipidemia (56.7%), CAD (28.4%), CVD (14.9%), CKD (23.9%), and DM (56.7%). Thirty-seven patients (55.2%) used angiotensin II receptor blocker for hypertension, and 7 (10.4%) patients used angiotensin converting enzyme inhibitor. Thirteen patients (19.4%) used *β*-blockers, 32 (47.8%) used statin, and 2 (3.0%) did nitroglycerin. The findings on laboratory tests are also shown in Table 1. With regard to hemodynamical parameters, the mean ABI level was 0.85 ± 0.18, which seemed to be relatively high, because we included the patients with PAD, which were treated by revascularization procedures, in this study. Furthermore, both of age and gender showed no statistically significant correlations with ABI values. The mean endothelial function level, measured by RHI using PAT, was 1.61 ± 0.51.

### 3.2. Correlations between the Endothelial Function and Other Parameters

The correlations between RHI and each of the parameters are shown in Table 2. None of the evaluated parameters, except for CVD and the ABI, showed any significant correlations with RHI in the univariate analysis. We divided the patients according to ABI values and categorized the patients with ABI (0.6–0.69) as group 1, ABI (0.7–0.79) as group 2, and ABI (0.8–0.89) as group 3. The RHI values were 1.29 ± 0.21 in group 1, 1.46 ± 0.49 in group 2, and 1.63 ± 0.73 in group 3. Furthermore, given that CVD (*P* = 0.143) and ABI (*r* = 0.271, *P* = 0.029) were defined as parameters correlated with RHI in the univariate analysis, we next evaluated the multivariate correlations between RHI and these parameters in Table 3. A multiple regression analysis revealed that the ABI significantly correlated with RHI (*β* = 0.254, *P* = 0.041) and it was also an independent and significant variable associated with endothelial function as measured by RHI.

## 4. Discussion

In this study, we examined the endothelial function in PAD patients using the PAT method. The endothelial dysfunction has been widely accepted as any form of abnormal activity of the endothelium. This dysfunction has been suggested to be associated with atherosclerotic disease and the risks of atherosclerosis [13–15]. The endothelial dysfunction may induce a wide range of proatherosclerotic molecular events, increase lipid permeability, and promote inflammation and oxidation within the atheroma. This stimulation of the atheroma might then lead to plaque rupture or prothrombotic events, such as those seen in CAD [3].

PAT is recorded as the change in the pulse wave amplitude, which can indicate microvascular function [16]. PAT reflects the direct contribution of nitric oxide, which has been shown to be strongly associated with endothelial function [17]. We therefore examined the correlation between the endothelial dysfunction measured by PAT and the incidence of atherosclerotic diseases, including the risk factors of atherosclerosis. However, we detected no significant associations between the endothelial dysfunction and the atherosclerotic-related diseases and factors. We might have obtained these inconclusive results because our study included a wide variety of patients with atherosclerotic comorbidities, with no obvious results obtained in the subgroup of PAD patients.

Although we were unable to detect any correlation between the endothelial dysfunction and atherosclerosis in the present study, we did reveal that the ABI was statistically correlated with the endothelial dysfunction measured using PAT. Brevetti et al. [18] reported that the ABI was significantly different between the patients with FMD > 6.2% and those with FMD < 6.2%. However, a few studies have reported an association between the endothelial function measured using the FMD method and outcomes in PAD patients, including ABI values [7, 19]. Allan et al. [20] reported that the PAT test did not show the significant difference between the patients with PAD and healthy subjects. However, some reports have shown any correlation between endothelial functions measured by PAT and the ABI [21]. Furthermore, Syvänen et al. [21] demonstrated the significant difference of RHI between normal ABI group and borderline ABI group. To our knowledge, this study is the first to demonstrate a significant and linear correlation between the ABI value and the endothelial dysfunction measured using PAT.

Though FMD might be promising for measuring endothelial dysfunction [6], we believe that PAT will show higher reproducibility than FMD, as the reproducibility of FMD depends on the technical skill of the ultrasound sonographer [22]. In addition to the correlation between PAT and the ABI value, we also demonstrated a correlation between the PAT test and CVD. Nezu et al. [23] reported that FMD < 4% was significantly correlated with CVD, including cerebral microbleeding. The findings from our study might support a significant correlation between PAT and CVD, as with FMD.

Several limitations associated with the present study warrant mention. First, this study had a small sample size, which might affect the statistical significance. Indeed, we noted no significant correlations between the endothelial dysfunction and atherosclerotic diseases, including CAD, which has shown a significant association with the endothelial dysfunction in other contexts [24]. Future studies should therefore be conducted in a larger population. Second, our population comprised PAD patients with relatively mild symptoms, such as intermittent claudication. The presence of PAD patients with critical limb ischemia or asymptomatic PAD patients in our population might have affected our findings. Despite these limitations, we feel that our results demonstrate a statistical correlation between the endothelial function and the ABI.

## 5. Conclusion

We herein demonstrated that the endothelial function measured by PAT significantly correlated with the ABI in PAD patients. However, the influence of several atherosclerotic-related conditions may have considerably affected these results. As such, the factors and conditions evaluated in the present study should be further examined in future studies in particular subgroups of subjects. In addition, further studies should be conducted to corroborate our findings regarding the endothelial function in patients with PAD and to confirm the PAT test as an appropriate method for determining the endothelial dysfunction of PAD patients.

## Figures and Tables

**Table 1:** Patients characteristics.

Variables	Subjects (*n* = 67)
Age (years)	73.6 ± 8.1
Gender (male/female)	50/17
Body mass index (kg/m^2^)	22.4 ± 2.5
Comorbidities	
Smoking history	51 (76.1%)
Hypertension	56 (83.6%)
Dyslipidemia	38 (56.7%)
Coronary artery disease	19 (28.4%)
Cerebrovascular disease	10 (14.9%)
Chronic kidney disease	16 (23.9%)
Diabetes mellitus	38 (56.7%)
Medications	
Nitroglycerin	2 (3.0%)
*β*-Blocker	13 (19.4%)
ACE-I	7 (10.4%)
ARB	37 (55.2%)
Statin	32 (47.8%)
Hemodynamical parameters	
Ankle brachial pressure index	0.85 ± 0.18
Reactive hyperemia index	1.61 ± 0.51
Laboratory findings	
White blood cell (/*μ*L)	6605 ± 1558
Hemoglobin (g/dL)	13.5 ± 1.9
Platelet (×10^4^/*μ*L)	22.6 ± 4.9
Prothrombin time (%)	99.6 ± 14.8
Activated partial thromboplastin time (sec)	29.7 ± 3.8
Fibrinogen (mg/dL)	323.1 ± 63.1
Albumin (g/dL)	4.02 ± 0.39
Creatinine (mg/dL)	1.07 ± 0.54
Total cholesterol (mg/dL)	191.9 ± 39.8
Triglycerides (mg/dL)	135.1 ± 123.2
High-density lipoprotein (mg/dL)	62.5 ± 22.6
Low-density lipoprotein (mg/dL)	110.2 ± 24.8
Hemoglobin A1c (%)	6.38 ± 0.74
C-reactive protein (mg/dL)	0.27 ± 0.48

ACE-I, angiotensin converting enzyme-inhibitor; ARB, angiotensin II receptor blocker.

**Table 2:** Univariate analysis of the correlations between endothelial function and various parameters.

Variables		*P* value
Categorical variables		
Gender		0.692
Smoking history		0.741
Hypertension		0.887
Dyslipidemia		0.972
Coronary artery disease		0.689
Cerebrovascular disease		0.143
Chronic kidney disease		0.316
Diabetes mellitus		0.667
Nitroglycerin		0.961
*β*-Blocker		0.860
ACE-I		0.191
ARB		0.972
Statin		0.475

	*r*	*P* value

Continuous variables		
Age	−0.020	0.986
Body mass index	−0.077	0.544
Ankle brachial pressure index	0.271	0.029
White blood cell	−0.071	0.573
Hemoglobin	−0.080	0.526
Platelet (×10^4^/*μ*L)	−0.045	0.725
Prothrombin time (%)	0.133	0.306
Activated partial thromboplastin time	0.146	0.263
Fibrinogen	0.105	0.438
Albumin	−0.040	0.760
Creatinine	0.149	0.236
Total cholesterol	−0.075	0.557
Triglycerides	−0.119	0.359
High-density lipoprotein	0.107	0.436
Low-density lipoprotein	−0.024	0.860
Hemoglobin A1c	0.085	0.516
C-reactive protein	0.011	0.930

ACE-I, angiotensin converting enzyme-inhibitor; ARB, angiotensin II receptor blocker.

**Table 3:** Multivariate analysis for the association of endothelial function with various parameters.

Variables	*β*	*t*	*P* value
Ankle brachial pressure index	0.254	2.092	0.041
Cerebrovascular disease	0.152	1.247	0.217
